# Quantitative evaluation of yeast's requirement for glycerol formation in very high ethanol performance fed-batch process

**DOI:** 10.1186/1475-2859-9-36

**Published:** 2010-05-21

**Authors:** Julien Pagliardini, Georg Hubmann, Carine Bideaux, Sandrine Alfenore, Elke Nevoigt, Stéphane E Guillouet

**Affiliations:** 1 Université de Toulouse, INSA, UPS, INP, LISBP, 135 Av de Rangueil, F-31077 Toulouse France INRA, UMR792 Ingénierie des Systèmes Biologiques et des Procédés, F-31400 Toulouse, France CNRS, UMR5504, F-31400 Toulouse, France; 2 Department of Molecular Microbiology, VIB, Kasteelpark Arenberg 31 - bus 2438, B-3001 Heverlee, Flanders, Belgium; 3 Laboratory of Molecular Cell Biology, Institute of Botany and Microbiology, Katholieke Universiteit Leuven, Kasteelpark Arenberg 31 - bus 2438, B-3001 Heverlee, Flanders, Belgium; 4 School of Engineering and Science, Jacobs University gGmbH, Campus Ring 1, 27579 Bremen, Germany

## Abstract

**Background:**

Glycerol is the major by-product accounting for up to 5% of the carbon in *Saccharomyces cerevisiae *ethanolic fermentation. Decreasing glycerol formation may redirect part of the carbon toward ethanol production. However, abolishment of glycerol formation strongly affects yeast's robustness towards different types of stress occurring in an industrial process. In order to assess whether glycerol production can be reduced to a certain extent without jeopardising growth and stress tolerance, the yeast's capacity to synthesize glycerol was adjusted by fine-tuning the activity of the rate-controlling enzyme glycerol 3-phosphate dehydrogenase (GPDH). Two engineered strains whose specific GPDH activity was significantly reduced by two different degrees were comprehensively characterized in a previously developed Very High Ethanol Performance (VHEP) fed-batch process.

**Results:**

The prototrophic strain CEN.PK113-7D was chosen for decreasing glycerol formation capacity. The fine-tuned reduction of specific GPDH activity was achieved by replacing the native *GPD1 *promoter in the yeast genome by previously generated well-characterized *TEF *promoter mutant versions in a *gpd2*Δ background. Two *TEF *promoter mutant versions were selected for this study, resulting in a residual GPDH activity of 55 and 6%, respectively. The corresponding strains were referred to here as *TEFmut7 *and *TEFmut2*. The genetic modifications were accompanied to a strong reduction in glycerol yield on glucose; the level of reduction compared to the wild-type was 61% in *TEFmut7 *and 88% in *TEFmut2*. The overall ethanol production yield on glucose was improved from 0.43 g g^-1 ^in the wild type to 0.44 g g^-1 ^measured in *TEFmut7 *and 0.45 g g^-1 ^in *TEFmut2*. Although maximal growth rate in the engineered strains was reduced by 20 and 30%, for *TEFmut7 *and *TEFmut2 *respectively, strains' ethanol stress robustness was hardly affected; i.e. values for final ethanol concentration (117 ± 4 g L^-1^), growth-inhibiting ethanol concentration (87 ± 3 g L^-1^) and volumetric ethanol productivity (2.1 ± 0.15 g l^-1 ^h^-1^) measured in wild-type remained virtually unchanged in the engineered strains.

**Conclusions:**

This work demonstrates the power of fine-tuned pathway engineering, particularly when a compromise has to be found between high product yield on one hand and acceptable growth, productivity and stress resistance on the other hand. Under the conditions used in this study (VHEP fed-batch), the two strains with "fine-tuned" *GPD1 *expression in a *gpd2*Δ background showed slightly better ethanol yield improvement than previously achieved with the single deletion strains *gpd1*Δ or *gpd2*Δ. Although glycerol reduction is known to be even higher in a *gpd1*Δ *gpd2*Δ double deletion strain, our strains could much better cope with process stress as reflected by better growth and viability.

## Background

Ideally, a micro-organism engineered for industrial biotechnology shows high product yield, final product concentration and productivity and can cope with process constraints. Achieving all these goals is a major challenge, particularly when it comes to modifications of the central carbon metabolism which is inherently coupled to energy and redox issues. Moreover, the cell's ability to cope with environmental stress can be severely affected. One prominent example for such a challenge is the reduction of glycerol formation in *Saccharomyces cerevisiae *(*S. cerevisiae*) in order to improve yield in ethanol production. Glycerol is produced from the glycolytic intermediate dihydroxyacetone phosphate (DHAP) which is reduced to glycerol-3-phosphate (G3P) by the two homologous isoenzymes of glycerol 3-phosphate dehydrogenase (GPDH), Gpd1 and Gpd2 [1,2]. G3P is then dephosphorylated into glycerol by the glycerol 3-phosphatases Gpp1 and Gpp2 [2,3]. In fact, glycerol is the main by-product beside carbon dioxide and biomass, accounting for up to 5% of the carbon [4].

A strain showing a reduced glycerol yield to the benefit of ethanol yield would result in substantial profit for the bioethanol industry. The challenge in reducing glycerol is that this compound and its formation fulfil major physiological functions in *S. cerevisiae*. Glycerol formation indeed participates in maintaining cytosolic redox balance [5-8] and in providing the intermediate G3P, essential for the biosynthesis of glycerophospholipids and triacylglycerols [9]. Glycerol is known to also contribute to stress management such as osmotic stress[10-12], heat, freezing/thawing or oxidative stress [10,13].

Although glycerol formation by wild-type *S. cerevisiae *is, to a certain extent, strain dependent, it predominantly depends on the environmental conditions. The most important environmental factors are oxygen availability, type of nitrogen source, osmotic pressure, heat and pH. For example, the presence of amino acids in the growth medium reduces the requirement of producing their carbon backbones. This results in a significantly lower generation of excess NADH, i.e. lower glycerol formation when compared to minimal medium without any amino acids [5].

The first studies which aimed at redirecting the carbon flux toward ethanol by reducing glycerol synthesis focused on GPDH (see above). Mutants deleted in one or both isogenes encoding for GPDH were constructed in different strain backgrounds and tested for ethanolic fermentation [1,3,7,14-17]. Nissen et al [16] reported that single deletion mutants *gpd1*Δ and *gpd2*Δ showed respectively a 2.8% and a 4.7% ethanol yield improvement under anaerobic conditions and a 2.2% and 3.3% under aerobic conditions. The double deletion mutant was not able to grow under anaerobic conditions and showed a 12.7% yield improvement, but also a 29% reduction in biomass yield in aerobic conditions. Other metabolic engineering strategies have targeted redox metabolism with the goal to produce less net excess NADH during the biomass synthesis and organic acid formation [18]. Bro et al. [19] obtained a 3% increase in the ethanol yield without any reduction in growth rate by by-passing the NAD^+^-dependent glycolytic conversion of glyceraldehyde to glycerate through the heterologous expression of a NADP^+^-dependent glyceraldehyde-3-phosphate dehydrogenase. Nevertheless, these studies were made on low glucose concentration and did not imply industrially relevant process stresses such as high glucose and ethanol concentration. It was indeed shown that a *gpd1*Δ *gpd2*Δ double deletion mutant was severely affected in ethanol production (35% decrease in final titre) and ethanol tolerance (25% reduction in the P_critical/μ _value (ethanol concentration at which growth stopped)) when placed under intensive ethanol production process[20].

Recent studies have combined single deletion of *GPD1 *or *GPD2*, engineering of redox metabolism and/or modification of yeast glycerol export [21-25] The best results, a 39.7% reduction in glycerol yield accompanied by a 12.3% ethanol yield improvement [25], were obtained with a strain deleted for *GPD1*, *FPS1 *and engineered for ammonium assimilation. Though, these results were obtained in a rich medium containing amino acids which is irrelevant in regards of most industrial processes. In addition, the use of rich medium compromises a correct conclusion about the redirection of carbon flow within these strains as long as catabolism of amino acids (in addition to glucose) is not taken into account.

Although some of the studies mentioned above gave interesting results, they suffer from a lack of information in terms of fermentative capacity in a high ethanol production process in which coping with process stress such as high ethanol titers becomes a critical issue for the yeast. For this purpose we previously developed a Very High Ethanol Performance fed-batch process [26] as a tool for studying the yeast physiology during ethanol production processes with high productivity and final ethanol concentration. We also showed that it was possible to reduce glycerol yield and final titer by finely monitoring the Respiratory Quotient (RQ) through glucose feeding in VHEP fed-batch [27]. However, this did not result in an increase in ethanol yield on glucose. We therefore searched for an avenue to further reduce glycerol formation in the VHEP fed-batch process. We envisaged identifying the optimal combination of process parameters and yeast strain genetic background for our process. However, it was clear from published data, that completely abolishing glycerol formation was accompanied by a drastic loss of process robustness [16,20]. We therefore envisaged an approach where glycerol formation capacity was strongly reduced but higher than in the *gpd1*Δ *gpd2*Δ double deletion strain. In this context, recent advances in yeast promoter engineering [28,29] have opened new possibilities for fine-tuning of metabolic fluxes. Based on the knowledge about Gpd1 and Gpd2 activities in yeast and available promoters for fine-tuning gene expression, an appropriate engineering strategy was defined supported by metabolic flux calculations (see results). To engineer the according strains, *GPD2 *was deleted and *GPD1 *expression was reduced by replacing its native promoter by two previously constructed *TEF1 *promoter versions [29] with strongly reduced but different activities. We present here the kinetic analysis of the two genetically modified strains by characterizing (i) the effect of the genetic modification on product formation and growth (rates, yields and titers) and (ii) the robustness of the strains in our VHEP fed-batch process.

## Methods

### Strains, media and growth conditions

The *Escherichia coli *strain DH5α™(Invitrogen Corp., Carlsbad) was used for amplification of plasmids. The strain was grown in Luria-Bertani (LB) medium (0.5% yeast extract, 1% peptone, 1% NaCl, pH 7) at 37°C. *E. coli *transformation and isolation of plasmid DNA were carried out using standard techniques [30]. All *Saccharomyces cerevisiae *strains used in this study are listed in Table 1 and were derived from the prototrophic haploid wild-type strain CEN.PK 113-7D. For initial pre-cultivations, yeast strains were grown on YPD plates (2 g L^-1 ^glucose, 1 g L^-1 ^yeast extract, 1 g L^-1 ^bacto peptone, 0.9 g L^-1 ^NaCl, 1.5 g L^-1 ^agar) and stored in 30% glycerol at -80°C. All yeast strains used in this study are prototrophic allowing the use of minimum mineral media without any amino acid supplementation. All subsequent pre-cultures and fermentation experiments were carried out in synthetic mineral medium prepared as follows (all concentrations in g L^-1^): KH_2_PO_4_, 3.0; (NH_4_)_2_SO_4_, 3.0; Na_2_HPO_4 _12H_2_O, 3.0; sodium glutamate, 1.0; MgSO_4 _7H_2_O, 0.5; ZnSO_4 _7H_2_O, 0.04; MnSO_4 _H_2_O, 0.0038; CoCl_2 _6H_2_O, 0.0005; CuSO4 5H_2_O, 0.0009; Na_2_MoSO_4 _2H_2_O, 0.00006; CaCl_2 _2H_2_O, 0.023; (NH_4_)_2_Fe(SO_4_)_6 _6H_2_O, 0.023; H_3_BO_3_, 0.003; pantothenate, 0.005; nicotinic acid, 0.005; meso-inositol, 0.125; thiamine, 0.005; pyridoxine, 0.005; para-aminobenzoic acid: 0.001, and biotin, 0.000012[31]. Three steps of propagation with increasing culture volumes (5 mL, 30 mL, 300 mL) were carried out before inoculating the reactor for the VHEP fed-batch fermentations. Each pre-culture was grown for 12 hours and used as the inoculum for the next step at a 10% v/v ratio.

**Table 1:** *Saccharomyces cerevisiae *strains used in this study

Strain	Genotype	Source or reference
CEN.PK 113-7D	*Wild Type*	van Dijken (2000) [52]
TEFmut7*	*gpd2Δ::loxP-ble*^*R*^*-loxP GPD1pΔ::loxP-KanMX4-loxP-TEF1p mutant 7*	This study
TEFmut2*	*gpd2Δ::loxP-ble*^*R*^*-loxP GPD1pΔ::loxP-KanMX4-loxP-TEF1p mutant 2*	This study

* These strains are isogenic to CEN.PK 113-7D.

### Engineered yeast strain construction

Genetic modifications of *S. cerevisiae *CEN.PK 113-7D carried out within this study comprise both the deletion of *GPD2 *and the replacement of native *GPD1 *promoter by two low-activity promoters (*TEF1 *promoters' versions see below). Gene deletion and promoter replacements based on homologous recombination in yeast were carried out according to the method described by Güldener *et al. *[32]. Transformation of *S. cerevisiae *was carried out according to Gietz *et al. *[33] using treatment with lithium acetate and polyethylene glycol. In order to allow expression of the antibiotic resistance genes cells directly after transformation were first incubated for at least 4 h at 30°C in YD containing 1% yeast extract and 1% glucose. Afterwards, cells were spread on YD agar plates supplemented with 7.5 μg/ml phleomycin (for *GPD2 *deletion) or 100 μg/ml geneticin G418 (for *GPD1 *deletion and integration of *GPD1 *promoter replacement cassettes).

*GPD1 *and *GPD2 *coding regions show strong similarities. In order to assure gene-specific homologous recombination of the *GPD2 *disruption cassette we used the *GPD2 *upstream region, which is different from the region upstream of *GPD1*. Primers used for amplification of the *GPD2 *disruption cassette and verification of correct gene disruption, listed in Table 2, were synthesized by Metabion International AG (Martinsried, Germany). Primers and PCR conditions used for the amplification of promoter replacement cassettes from our *TEF1 *promoter mutant collection were the same as previously described [29]. The thermostable *Pfu *DNA polymerase with proofreading activity was obtained from BIONEER (Korea) and used for amplification of both gene-disruption and promoter-replacement cassettes. Top DNA polymerase (BIONEER, Korea) was used in all diagnostic PCRs. PCR reaction mixtures were prepared according to the manufacturer's guidelines.

**Table 2:** Primers used for amplification the *GPD2 *disruption cassette and verification of its correct genomic integration

**No**.	Function	Sequence
P60*	Forward primer for amplification of *GPD2 *deletion cassette	5'-TAGCTTACGGACCTATTGCCATTGTATTCCGATTA ATCTATTGTcagctgaagcttcgtacgc-3'
P61*	Reverse primer for amplification of *GPD2 *deletion cassette	5'-CACATTCTCACCTCTGGCTCGAAGATATGGGAATGCAATTCTGTgcataggccactagtggatctg-3'
P62	Forward primer for verification of *GPD2 *deletion	5'-ACGATGG CTCTGCCATT-3'
P63	Reverse primer for verification of *GPD2 *deletion	5'-GATCAGGATCGGCCACTA-3'

*Sequences in low chase letters correspond to the primer sequences for the amplification of the *loxP-bleR-loxP *deletion cassette from the plasmid pUG66 used as a template [32]. Nucleotides in capital letters are derived from *S. cerevisiae *S288c genome sequence for integration of the deletion cassette by homologous recombination at the *GPD2 *locus.

The *GPD2 *gene was deleted using the *loxP-ble*^*R*^*-loxP *cassette located on the plasmid pUG66 [32]. The gene *ble*^*R *^confers resistance to phleomycin. The primers used for the amplification of the disruption cassette were P60 and P61 (Table 2). The *GPD2 *disruption cassette used here replaced 305 bp upstream of the *GPD2 *coding region and 360 bp of the *GPD2 *coding sequence. The correct integration of the *loxP-ble*^*R*^*-loxP *cassette was verified by diagnostic PCR using the primer pair P62/P63 (Table 2) and the following PCR conditions: 94°C for 1 min, 50°C for 1 min, and 72°C for 2 min. The PCR was performed in 30 cycles. If wild-type genomic DNA was used as a template, this diagnostic PCR resulted in a product of 855 bp in size, whereas the product obtained from positive *gpd2*Δ transformants had a size of 1.5 kbp.

In order to replace the native *GPD1 *promoter by promoters of much lower activities, the *TEF1 *promoter mutant versions 2 and 7 of our previously published promoter collection for fine-tuning gene expression in yeast [29] were used. The promoters were located on the described CEN/ARS plasmids *p416-loxP-KmR-TEFmut2-yECitrine *and *p416-loxP-KmR-TEFmut7-yECitrine *bearing the *loxP-KanMX-loxP *cassette upstream of the *TEF1 *promoter mutant 2 and 7, respectively. Integrations of the low-strength promoters were confirmed by PCR diagnosis using primers and PCR conditions as described earlier [29] except the temperature for primer annealing was set to 57°C instead of 60°C. The PCR product obtained from native *GPD1 *promoter was 1.6 kbp, while a positive integration of the *TEF1 *promoter mutant 2 or 7 cassette yielded a product size of 2.6 kbp.

### Measurement of specific GPDH activity

In order to determine the specific activity of glycerol 3-phosphate dehydrogenase (GPDH), yeast strains were aerobically grown in shake flask cultures using the synthetic minimal medium as described above supplemented with 2% [w/v] glucose. The GPDH activity was measured in logarithmically growing cells (i.e. when OD_600 _reached about 1) according to a previously described method [2,34].

### VHEP fed-batch protocol

VHEP fed-batch fermentations were carried on in 5 L bioreactors B DCU B.BRAUN (SARTORIUS) with a starting volume of 3 L. Temperature was set at 30°C and pH regulated at 4 by adding 14% (vol/vol) NH_3 _solution. The reactor was flushed continuously with air; dissolved O_2 _was maintained above 20% of saturation by adapting the air flow and stirring rate in order to maintain fully aerated conditions. A sequential vitamin feeding strategy based on the growth profile [31] was applied. The fermentations were started with an initial glucose concentration of 100 g L^-1^. Whenever the residual glucose concentration was lower than 20 g L^-1^, glucose feeding was carried out with a 700 g L^-1 ^glucose solution to restore a glucose concentration of 100 g L^-1^. At the later phase of fermentation, i.e.when the ethanol concentration was above 90 g L^-1^, the glucose feeding adjusted the concentration to 50 g L^-1^.

### Gas analysis

Outlet and inlet gas analysis was performed using a mass spectrometer Proline Dycor^2^a (Ametek Process Instrument). Gas analysis was performed on the outlet flow of the reactor every 5 minutes and on the inlet air every hour. The volumetric O_2 _consumption rate and the CO_2 _production rate were calculated from the differences between the inlet and outlet gas compositions, taking into account the evolution of the liquid volume in the reactor, the inlet airflow rate (regulated by a mass flowmeter), the temperature and the pressure.

### Analytical methods

Yeast growth was evaluated by spectrophotometric measurements at 620 nm in a spectrophotometer Libra S4 (Biochrom) and calibrated against cell dry weight measurements. Cells were harvested by filtration on 0.45-μm-pore-size polyamide membranes (Sartorius Biolab Product) and dried to a constant weight at 60°C under a partial vacuum (200 mm Hg ~ 26.7 kPa) for 24 hours. Rapid determination of glucose and ethanol concentrations from broth supernatants during fermentation was performed with an YSI analyser (YSI model 27 A; Yellow Springs Instruments).

Determination of ethanol, organic acids and glucose from supernatants was performed by HPLC using an Aminex HPX-87H+ column (300 mm × 7.8 mm) and dual detection (refractometer and UV at 210 nm) at 50°C with 5 mM H_2_SO_4 _as an eluant (flow rate of 0.5 mL min^-1^). Three independent metabolite quantifications (taking into account sampling, separation and HPLC quantification) showed that the measurement was reproducible; typically standard deviation was lower than 1% of mean value for ethanol and glucose and lower than 5% for glycerol and acetate.

### Chemicals

All chemicals were of the highest analytical grade available.

### Determination of the cells' viability

To determine cells' viability, the methylene blue technique was used as previously described [31].

### Assessment of ethanol evaporation

When balancing aerobic ethanolic fermentation experiments, there is commonly a lack in carbon due to evaporation of ethanol. In fact, this may account for more than 20% of the total carbon [16,35]. To assess the part of evaporated ethanol, evaporation experiments were carried out under the same cultivation conditions as performed in this study but without yeast. The bioreactor was filled with 3 liters of the synthetic mineral medium used in this study and ethanol was added up to a concentration of 150 g L^-1^. Decrease in the ethanol concentration was recorded over time by taking regular liquid samples and HPLC quantification. The rate of evaporation was found to be dependent on ethanol concentration, aeration rate and liquid volume. No significant impact of stirring on evaporation was observed which is in accordance to a previous study [35]. Evaporation was investigated for the 2 aeration rates used during the fermentations 1 L.mn^-1 ^and 0.5 L.mn^-1^. Based on these data, an evaporation rate was attributed to each ethanol liquid concentration. Integration of this evaporation rate over time and liquid volume allowed calculating the evaporated ethanol in our yeast fermentations. The fact that the measured ethanol evaporation during the period of fermentation when cells had stopped producing ethanol exactly matched the calculated ethanol evaporation validated the method.

### Metabolic Flux Calculation

Metabolic fluxes were calculated using a MFA based model extrapolated from a previously described Metabolic Descriptor [27,36] The metabolic network was modified by adding mitochondrial compartmentation. Repartition of the reactions between cytosol and mitochondria was made according literature and previously described models [37-42]. Glycolysis, pentose phosphate pathway, synthesis of amino-acid precursors and nitrogen bases as well as formation of ethanol and glycerol were attributed to the cytosol. TCA cycle and respiratory chain were attributed to the mitochondria. Acetate and acetyl-coA synthesis were assumed to be localized in both cytosol and mitochondria. Segregation of amino acids synthesis between cytosol and mitochondria was inserted into the model according to the literature cited above. All transport reactions between the cytosol and the mitochondria were assumed to be simple transport reactions except for redox equivalent translocation via the glycerol shuttle. Our final metabolic network consists in 142 reactions, including 14 exchange reactions between the cell and external medium, 88 cytosolic reactions, 24 transport reactions between mitochondria and cytosol, and 16 mitochondrial reactions (cf. Additional file 1). Validity of the model was assessed using published data based on ^13^C labelling experiments [43,44].

The Yatp,x values and NADH balance were calculated from the results of metabolic flux calculation as follows:

: Stoichiometric coefficient of metabolite x in reaction i

: Rate of reaction i belonging to metabolic pathway y

*Ana: *Anabolism

*Gly*: Glycolysis

*OP*: Oxidative Phosphorylation

*TCA*: Tricarboxylic acid cycle

*Glyce*: Glycerol

*Etoh*: Ethanol

*NADHcon*: NADH consumed

*NADHpro*: NADH produced

The uncertainties of the TCA cycle fluxes values were estimated to impact the Yatp,x and NADH balance by less than 3%.

## Results

### Design and construction of the *GPD *engineered strains supported by *in silico *flux calculations

**Figure 1:**
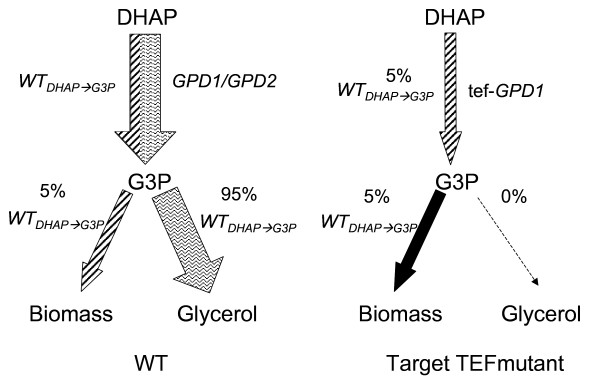
**Flux repartition into the glycerol pathway**. Fluxes were calculated using our Metabolic Descriptor model (see Methods, see Additional file 1) and experimental data obtained from a VHEP fed-batch fermentation with the wild type strain CEN.PK 113-7D.

The central question of this study was to evaluate whether and to what extent glycerol formation in *S. cerevisiae *can be reduced without severely affecting yeast's robustness, particularly ethanol tolerance under Very High Ethanol Performance fed-batch process conditions. Apart from the knowledge about the remarkably reduced ethanol tolerance of the *gpd1*Δ *gpd2*Δ double deletion strain, no quantitative data was available about the impact of reduced glycerol formation capacity on yeast's performance in our VHEP fed-batch process. A data set of specific substrate consumption and metabolite production rates measured during VHEP fed-batch cultivation with the CEN.PK 113-7D wild-type strain allowed us to calculate intracellular carbon fluxes including the one from DHAP to G3P necessary to fulfil biomass building block requirements. Flux calculations revealed that the ratio between the specific DHAP-to-G3P conversion rate and the specific growth rate were constant over a long period of the growth. Based on our metabolic model, 5% of the total DHAP-to-G3P flux observed in the wild-type strain was required for anabolic reactions while 95% of this flux was diverted toward glycerol production (Figure 1). This 5% flux should represent the minimum of carbon flux needed for anabolic requirements. Thus, one obligation for strain construction was to provide them with this minimal glycerol formation capacity.

In order to engineer *S. cerevisiae *strains with a strongly reduced but not completely abolished glycerol formation capacity, we envisaged to delete one isogene of GPDH and fine-tune the expression of the other one by replacing its native promoter by another one with much lower activity. We decided to delete *GPD2 *and fine-tune *GPD1 *expression since the latter is known to be responsible for the major part of GPDH activity and *GPD2 *deletion has been demonstrated to have no major impact on yeast physiology except when synthetic minimal medium was used under strictly anaerobic conditions [45]. In order to replace the native *GPD1 *promoter in CEN.PK 113-7D *gpd2*Δ background, two previously characterized mutated versions of the *S. cerevisiae TEF1 *were used (see Material and Methods). We chose the two weakest promoters available for this study, i.e. *TEF1*p mutant 2 (normalized promoter strength 7%) and *TEF1*p mutant 7 (promoter strength 16% normalized to the native *TEF *promoter) [29]. Specific GPDH activity of the wild type was 0.041 U/mg protein. The down-regulation of *GPD1 *gene expression by *GPD1 *promoter replacement in the *gpd2*Δ background resulted in significantly reduced GPDH activities. The use of *TEF1*p mutant 7 upstream of *GPD1 *reduced GPDH activity to 55% (0.023 U/mg protein) whereas the use of *TEF1*p mutant 2 reduced GPDH activity to 6% (0.006 U/mg protein) compared to wild-type activity.

### Impact of reduced GPDH activity on fermentations parameters

**Figure 2:**
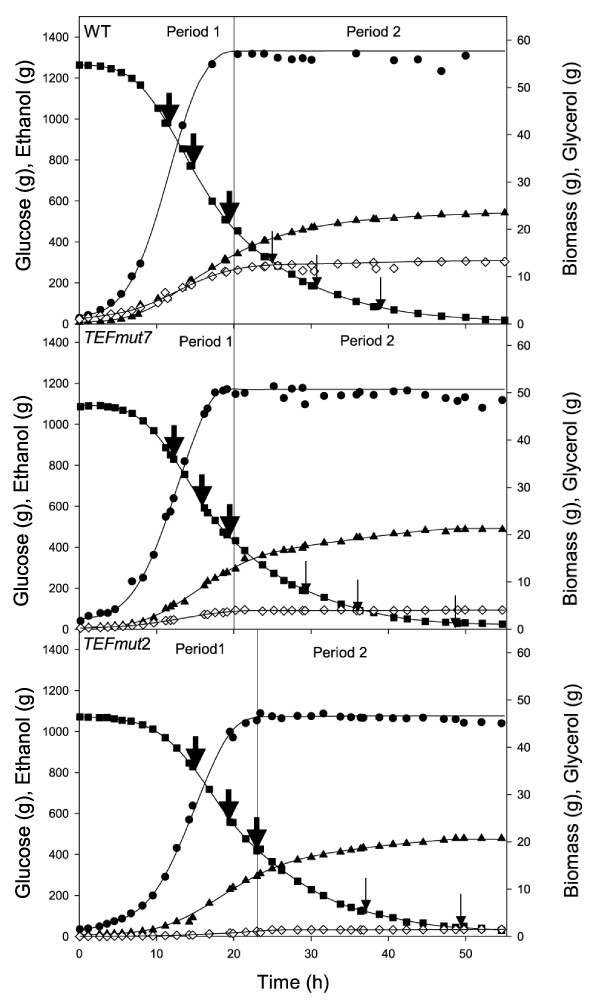
**Substrate and products masses evolutions during the wild type and the mutants fermentations**. Mass of glucose (black square), ethanol (black triangle), biomass (black circle) and glycerol (white diamond). Period 1 corresponds to the "growth/production phase" and period 2 to the "production phase". Thick arrows indicate the time points when glucose feeding was carried out in order to adjust glucose concentration in the fermenter up to 100 g L^-1^, thin arrows correspond to glucose feeding up to a concentration of 50 g L^-1^.

The two engineered strains *TEFmut7 *and *TEFmut2 *and the wild type were studied in VHEP fed-batch cultivation under comparable operating conditions in a synthetic mineral medium in order to precisely quantify the effect of the modulation of glycerol synthesis on ethanol production and growth capacities. Time courses of glucose consumption, biomass, ethanol and glycerol production are shown in Figure 2. All three fermentations showed two characteristic phases: a first "growth phase" where biomass was produced concomitant with ethanol, and a second "production phase", where growth had stopped due to ethanol inhibition but cells kept on producing ethanol. Growth of both the wild-type strain and the *TEFmut7 *strain ended after about 20 hours while growth of *TEFmut2 *ended after 23 hours.

Calculated growth rate, biomass yield, glycerol yield as well as ethanol yield, final titer and productivity are summarized in Table 3. Carbon balances were closed to 89% for the wild type, to 94% for *TEFmut7 *and to 94% for *TEFmut2*; the degree of reduction balances closed to 83%, 90% and 90%, respectively. Evaluation of evaporated ethanol resulted in carbon and degree of reduction balances above 94% for all three fermentations. Acetaldehyde was not measured due to its volatility and could likely explain part of the deficit in carbon and degree of reduction balances.

**Table 3:** Fermentation characteristics for *S. cerevisiae *wild-type strain CEN.PK 113-7D and the two mutants

	WT	*TEFmut7*	*TEFmut2*
**Fermentation Time (h)**	55	49	49
**Growth Time (h)**	20	20	23
**Final Volume (L)**	3.10	2.89	2.92
**μ**_**max **_**(h**^**-1**^**)**	0.35 ± 0.02	0.28 ± 0.02	0.24 ± 0.02
**DCWmax (g L**^**-1**^**)**	15.7 ± 0.5	14.5 ± 0.5	14.5 ± 0.5
**YDCW/glucose g g**^**-1**^	0.093 ± 0.006 *[5-15 h]*	0.091 ± 0.007 *[5-15 h]*	0.087 ± 0.005 *[5-17 h]*
**Yethanol/glucose g g**^**-1 **^**(growth)**	0.41 ± 0.006 *[0-20 h]*	0.42 ± 0.01 *[0-20 h]*	0.44 ± 0.005 *[0-23 h]*
**Yethanol/glucose g g**^**-1 **^**(overall)**	0.43 ± 0.005 *[0-55 h]*	0.44 ± 0.008 *[0-49 h]*	0.45 ± 0.003 *[0-49 h]*
**Y glycerol/glucose mg g**^**-1**^	13 ± 0.01 *[0-20 h]*	5.1 ± 0.2 *[0-20 h]*	1.6 ± 0.1 *[0-23 h]*
**[ethanol] final (g L**^**-1**^**)**	117 ±4	112 ± 4	114 ± 4
**[glycerol] final (g L**^**-1**^**)**	3.1 ± 0.1	1 ± 0.1	0.4 ± 0.1
**Ethanol Productivity (g L**^**-1 **^**h**^**-1**^**)**	2.13 ± 0.15	2.2 ± 0.15	2.3 ± 0.15
**Pcritical/μ (g L**^**-1**^**)**	87 ± 3	85 ± 3	86 ± 3

μ_max_: maximum specific growth rate; DCW_max_: maximum cell concentration; Yi/glucose: production yield of the constituent i on glucose; Pcritical/μ: ethanol concentration at which growth stopped; "growth phase": fermentation phase of yeast growth; "production phase": fermentation phase after yeast growth stopped but ethanol production continued.

The final biomass concentration obtained for the wild-type strain was 15.7 g L^-1 ^while both modified strains showed a final biomass concentration of 14.5 g L^-1^. Final glycerol concentration was 3.1 g L^-1 ^for the wild-type strain compared to 1 g L^-1 ^for *TEFmut7 *and 0.4 g L^-1 ^for *TEFmut2*. The final ethanol concentration reached was 117 g L^-1 ^for the wild-type and slightly reduced to 112 g L^-1 ^for *TEFmut7 *and 114 g L^-1 ^for *TEFmut2*. At the end of fermentation, acetate concentration reached 5.5 g L^-1 ^in the wild type and 5.1 g L^-1 ^in the two mutants.

### Impact of reduced GPDH activity on fermentation kinetic parameters

The reduction of GPDH activity in the engineered strains led to a decrease in the maximum specific glycerol production rate from 0.083 g g_DCW _h^-1 ^in the wild type to 0.023 g g_DCW _h^-1 ^and 0.004 g g_DCW _h^-1 ^in *TEFmut7*and *TEFmut2*, respectively. This corresponds to 28 and 5% residual rates in *TEFmut7*and *TEFmut2 *compared to the wild type, respectively. However, the maximum specific growth rate and the maximum specific ethanol production rates were also reduced in the strains engineered for lower GPDH activity. The μ_max _was 0.35 h^-1 ^for the wild type, 0.28 h^-1 ^for *TEFmut7 *and 0.24 h^-1 ^for *TEFmut2*. The maximum specific ethanol production rates were 1.31 g_ethanol _g_DCW_^-1 ^h^-1 ^in the wild type, 1.10 g_ethanol _g_DCW_^-1 ^h^-1 ^in *TEFmut7 *and 0.98 g_ethanol _g_DCW_^-1 ^h^-1 ^in *TEFmut2 *corresponding to 16% and 25% reduction in the strains *TEFmut7 *and *TEFmut2*, respectively. Nevertheless, the overall volumetric ethanol productivity was hardly affected in *TEFmut7 *and *TEFmut2, i.e. *2.2 ± 0.1 and 2.3 ± 0.1 g L^-1^h^-1^, respectively compared to 2.1 ± 0.15 g L^-1^h^-1 ^for the wild type.

### Impact of reduced GPDH activity on yields

Glycerol yield based on consumed glucose was 0.0051 g g^-1 ^and 0.0016 g g^-1 ^for *TEFmut7 *and *TEFmut2*, respectively, corresponding to 39% in *TEFmut7 *and 12% in *TEFmut2 *compared to the wild type strain (0.013 g g^-1^). The glycerol yields per g of biomass during the growth phase were also much lower for *TEFmut7 *and *TEFmut2 *(i.e. 0.06 and 0.02 g g^-1^_DCW_) respectively, compared to 0.14 g g^-1^_DCW _for the wild type.

The biomass yields on glucose in *TEFmut7 *and *TEFmut2 *were slightly lower in the strains with the reduced GPDH activity, i.e. 0.091 g_DCW _g_glucose_^-1 ^for *TEFmut7 *and 0.087 g_DCW _g_glucose_^-1 ^*TEFmut2 *compared to 0.093 g_DCW _g_glucose_^-1 ^for the wild type. The overall acetate yields based on consumed glucose were similar for all three strains and reached 0.017 g g^-1^.

As a result, the overall ethanol production yield on glucose was increased in the two engineered strains. While the wild-type strain produced 0.43 g g^-1^, *TEFmut7 *formed 0.44 g g^-1 ^and *TEFmut2 *0.45 g g^-1^. As visible from Table 3, the strain differences in the overall ethanol yields were solely due to the differences observed during the growth phase. In this phase, ethanol yields reached 0.41 g g^-1 ^for the wild type, 0.42 g g^-1 ^for *TEFmut7 *and 0.44 g g^-1 ^for *TEFmut2*. During the "production phase", no significant variation of ethanol yield could be observed between the wild type and the two engineered strains (0.47 g g^-1^).

We also calculated oxygen to biomass yield for all three strains. The average oxygen to biomass yield during the growth phase of the wild type was 6 mmol_O2 _g_DCW_^-1^. While there was no significant difference compared to *TEFmut7 *(7 mmol_O2 _g_DCW_^-1^), the strain *TEFmut2 *showed a significantly higher yield of 10 mmol_O2 _g_DCW_^-1^.

### Impact of reduced GPDH activity on ethanol tolerance

**Figure 3:**
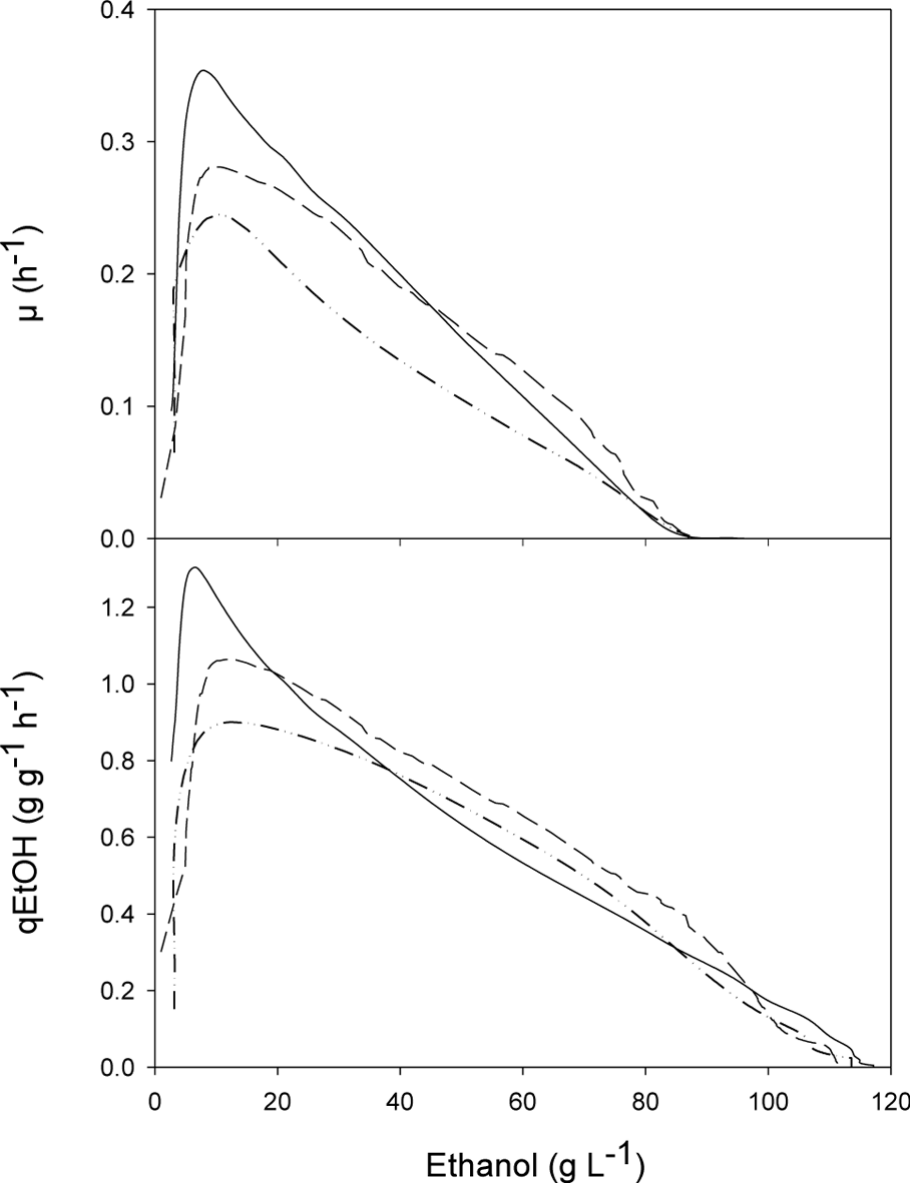
**Specific growth rates (μ) and specific ethanol production rate (qEtOH) as functions of ethanol concentration**. Strains: wild type CEN.PK 113-7D (___), *TEFmut7 *(- - -), *TEFmut2*. (--- ..).

**Figure 4:**
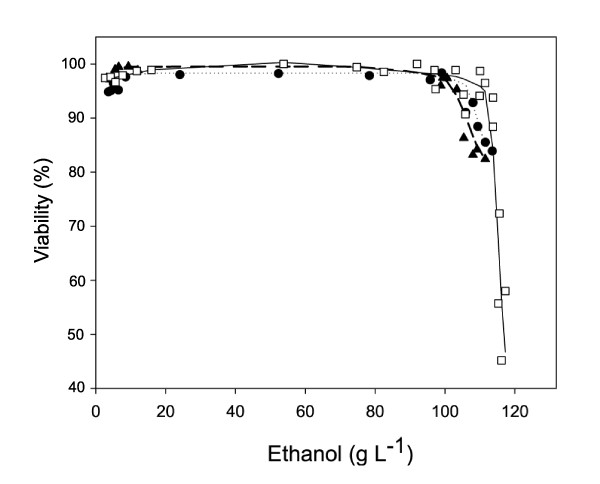
**Cell viability as a function of ethanol concentration**. Strains: wild type CEN.PK 113-7D (white square), *TEFmut7 *(black triangle), *TEFmut2 *(black circle).

Figure 3 shows the evolution of both the specific growth and ethanol production rates as a function of the actual ethanol concentration in the bioreactor for each strain. The ethanol concentration at which uncoupling between growth and ethanol production occurs is usually referred to as Pcritical/μ. This parameter characterizes the strain-dependent growth inhibition by ethanol. Pcritical/μ was about 87 g L^-1 ^for the wild type, 85 g L^-1 ^and 86 g L^-1^for *TEFmut7 *and *TEFmut2*, respectively. Evaluation of cell viability by the methylene blue method indicated that ethanol tolerance of the *GPD*-engineered strains was only slightly reduced compared to the wild type (Figure 4).

### Comparative flux analysis of the strains

**Figure 5:**
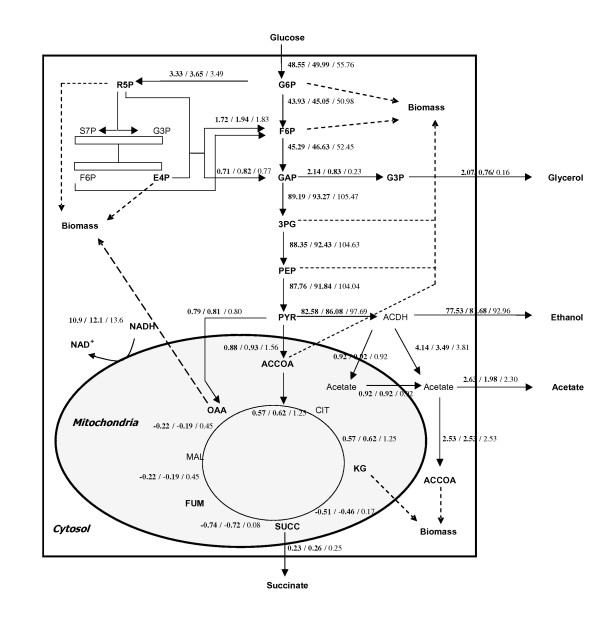
**Metabolic flux repartition**. Metabolic flux repartition within the central carbon metabolism for the *S. cerevisiae *CEN.PK 113-7D wild type and the two strains engineered for reduced GPDH activity. Metabolic fluxes were calculated as mmol g_DCW_^-1 ^h^-1 ^from experimental data obtained at μ_max _and then normalized to a biomass production rate of 1 g g^-1 ^h^-1 ^(**WT**/***TEFmut7***/*TEFmut2*). TCA cycle fluxes were found highly sensitive to low variation of qetoh/μ ratio and therefore should be taken with caution.

In order to better understand the metabolic reorganization upon the modulation of the glycerol synthesis pathway at the cell level, metabolic flux calculations were carried out for each strain. However in order to take into account the differences in μ_max _between the strains, the experimentally obtained specific consumption and production rates were chosen at μ_max _and normalized to a biomass production rate of 1 g g^-1 ^h^-1^. The results of this calculation are reported in Figure 5. It shows that the DHAP-to-G3P flux at μ_max _was only 39% and 11% in *TEFmut7 *and *TEFmut2*, respectively compared to the level observed in the wild type. Moreover flux calculation also indicated that the modulation of the glycerol pathway led to a global metabolic reorganization pointed out by the increased normalized rates of ethanol production, glycolysis, NADH mitochondrial shuttles and respiration.

## Discussion

Aiming at determining to what extent glycerol formation may be reduced without affecting drastically the strain robustness in a VHEP fed-batch process, the modulation of glycerol synthesis capacity of *S. cerevisiae *was obtained by replacing the native promoter of *GPD1 *with promoters of significantly lower activities in a CEN.PK113-7D *gpd2*Δ background resulting in the strains *TEFmut7 *and *TEFmut2*.

### Fine-tuning of the glycerol synthesis pathway led to improved ethanol yield

The two engineered strains led to a reduction of glycerol yield on glucose by 61% for *TEFmut7 *and 88% in *TEFmut2 *compared to the wild type strain. The metabolic flux calculation from the experimental data set of *TEFmut7 *and *TEFmut2*, respectively, showed that the DHAP-to-G3P flux was evaluated at 39 and 11% of the one calculated in the wild type (Figure 5). The reduction of the glycerol production in the two engineered strains was accompanied by a slightly increased ethanol yield on glucose (2.3% for *TEFmut7 *and 4.6% for *TEFmut2*). For comparison, in aerobic conditions, simple deletion mutants *gpd1*Δ and *gpd2*Δ of TN1 strain showed respectively a 2.2% and 3.3% yield improvement, whereas double deletion *gpd1*Δ*gpd2*Δ strain showed a 12% improvement (0.39 g g^-1^) [16]. A 10% ethanol yield improvement was also obtained in a double deletion *gpd1*Δ *gpd2*Δ strain of W303-1A but was accompanied by a drastic loss of robustness towards ethanol stress [20].

**Figure 6:**
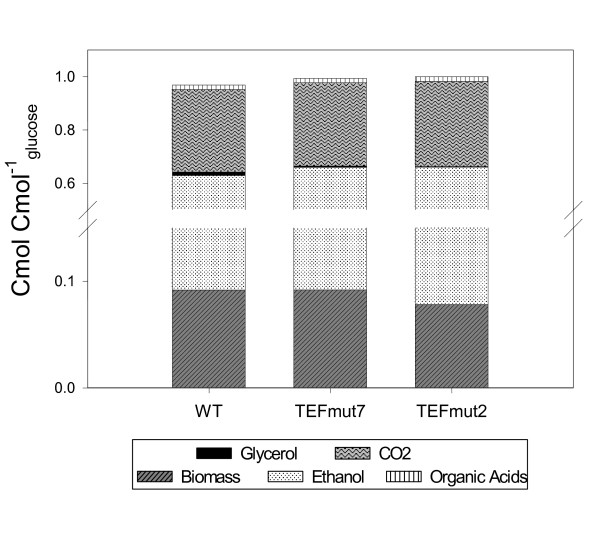
**Carbon balances for the *S. cerevisiae *wild-type strain and the two mutants**. Carbon balances and are expressed as C-mol ratio between the amount of metabolites produced (based on final masses at the end of fermentation) and glucose consumed.

In terms of carbon balance (Figure 6), the reduction of the glycerol production could not completely explain the gain in the ethanol yield. Obviously, a decrease in biomass production also contributed to this improvement. Metabolic flux calculations pointed out a decrease in the ATP-to-biomass yield (Yx,ATP) concomitantly with the decrease in the biomass production yield. The Yx,ATP value was 9.7, 9.1 and 7.8 g_DCW _mol_ATP_^-1^, in the wt , the *TEFmut7 *and the *TEFmut2*, respectively. A decrease in the ATP yield related to the decrease in glycerol production was already observed in RQ controlled fermentation experiment [27]. This diminution of the biomass and ATP yield could be linked to the deficit of cytosolic NADH oxidation (discussion see below) and to the stress management. The cell stress management may cost more energy to the mutant strains under VHEP conditions due to high glucose and ethanol concentrations and high osmolarity (rise from 0.7 to 2.9 Osm kg^-1 ^during the whole cultivation). For transport systems energetically related to the proton gradient, the cost in "equivalent" ATP may be higher in the strains producing less glycerol, known as the main compatible solute in yeast. For instance, osmoregulatory mechanisms such as K^+ ^homeostasis, glycerol exporter (FPS1) and compatible solute synthesis requiring appreciable ATP turnover [46] may be more requested in strains impaired in glycerol formation. Metabolic flux calculation showed that the mutant strains generated more energy than the wt to produce a similar amount of biomass (Figure 5) through a higher flux in the central carbon metabolism. Higher maintenance coefficients and higher TCA cycle flux were already reported in the literature during osmoregulation in *S. cerevisiae *[46,47].

### Fine-tuning of the glycerol synthesis pathway altered the growth rate but not the ethanol tolerance in VHEP

**Figure 7:**
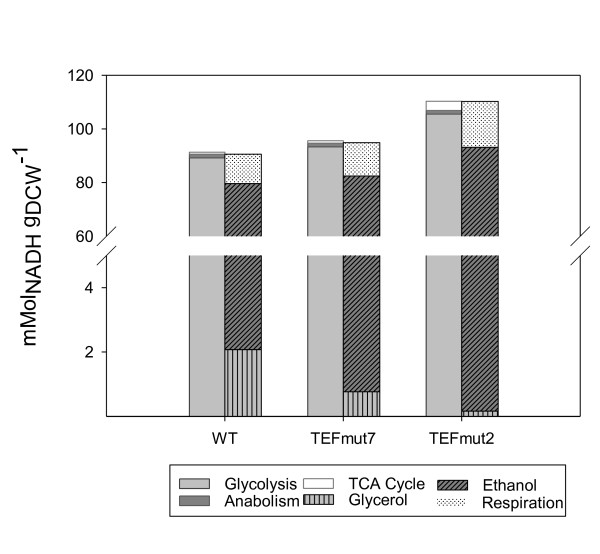
**NADH balances for the *S. cerevisiae *wild-type and the two mutants (*TEFmut7 *and *TEFmut2*)**. NADH balances were calculated for each strain at the time point when μ_max _was reached, respectively. Left bars correspond to generated NADH (by glycolysis, anabolism and TCA cycle); right bars correspond to the oxidized NADH (by glycerol and ethanol formation pathways and respiration).

The reduction of the glycerol formation in the two mutant strains led to a concomitant decrease in the maximal specific growth rate (μ_max_), 20% and 31% lower in the strains *TEFmut7 *and *TEFmut2*, respectively, comparatively to the wt strain. Nevertheless, based on the metabolic flux calculation, the DHAP-to-G3P rate should have been high enough for both strains to sustain the μ_max _of the wild type. Similarly a 55% decrease in the μ_max _was reported in *gpd1*Δ *gpd2*Δ strain under aerobic conditions, explained by the limited ability to reoxidize NADH to NAD^+ ^in the cytosolic compartment [16]. The deficit of cytosolic NADH oxidation due to the decrease in glycerol formation capacity could be partly compensated by the increase in the oxygen consumption through the respiration, observed in the mutant strains. Figure 7 indeed shows the increased participation of ethanol biosynthesis and respiration to the NADH balance. The metabolic flux calculation revealed also an increased participation of the mitochondrial shuttles.

The mitochondrial shuttles were represented in the model by the glycerol-3-phosphate shuttle, but either an increased participation of the external NADH dehydrogenases (*NDE1/2*) or the glycerol-3-phosphate shuttle could take over the excess of cytosolic NADH [8,48]. Therefore both systems likely participated to the transfer of cytosolic NADH into the mitochondria to be oxidised by the respiratory chain leading to the increase in the O_2 _consumption. However the reduction of the GPDH rate may have reduced the activity of the glycerol-3-phosphate shuttle compared to the NDEs in our engineered strains. As the glycerol-3-phosphate shuttle was reported to have a higher ATP/O ratio compared to the external NDEs, the reduction of the glycerol-3-phosphate shuttle activity could partly explain the reduction in Yx,ATP in our mutant strains [8].

One alternate explanation for growth rate reduction might be that the reduction in the glycerol phosphate dehydrogenase rate created a competition for the L-G3P between the anabolic requirement in this metabolite and glycerol synthesis within the cell. L-G3P produced by the reduction of DHAP is indeed either dephosphorylated into glycerol by the glycerol phosphate phosphatases (GPP) or enters into the phospholipid synthesis pathway via the phosphatidic acid (Figure 1). The rate of dephosphorylation of L-G3P being far higher than the rate of its conversion into phosphatidic acid, this latter reaction might limit the growth rate.

No relevant reduction of the maximum specific growth rate was observed in single *gpd*Δ deletion mutants in previous studies in aerobic conditions [16]. However ethanol production capacities of these strains were studied under experimental conditions where the cells did not face high glucose and high ethanol concentration as encountered in VHEP conditions. Glycerol is known to play a role in stress resistance, such as osmotic stress [10,49], ethanol and temperature stress [13]. Glycerol is the major compatible solute accumulated in yeast to increase cell turgor pressure to face hyper-osmotic stress. Modifying the glycerol synthesis pathway should alter the ability of the cell to regulate its turgor pressure. However authors have suggested through the surface stress theory that a fine tuned turgor pressure is necessary for yeast growth [50,51]. Thus an impact of the osmotic stress on the μ_max _of our strains under our conditions cannot be completely excluded.

Despite the impact on the growth rate and in clear contrast to the *gpd1*Δ*gpd2*Δ double deletion strain [20], ethanol tolerance was not affected by the genetic modifications carried out in the current study even though the fermentation conditions were exactly the same.

Finally we concluded that the two mutant strains were able to sustain a similar ethanol concentration than the wild type strain but with a higher energy expense. This higher energy demand increased the relative weight of energy production reactions over biomass synthesis in the metabolism of the mutants leading to an improved ethanol yield.

## Conclusions

A prominent feature here is that fine-tuning the glycerol synthesis pathway (within the range 11-39% of the wild-type capacity) allows the strains to keep their initial ethanol tolerance. Both engineered strains showed indeed a similar behaviour in terms of ethanol inhibition on growth quantified by a Pcritical/μ value at 86 ± 1 g L^-1 ^and viability. Therefore we conclude that reducing the glycerol synthesis down to 11% of the wild type capacity did not affect the strain robustness in terms of ethanol tolerance, ethanol titer and productivity.

## Abbreviations

: Stoichiometric coefficient of metabolite x in reaction i; : Rate of reaction i belonging to metabolic pathway y; *Ana: *Anabolism; *Gly*: Glycolysis; *OP*: Oxidative Phosphorylation; *TCA*: Tricarboxylic acid cycle; *Glyce*: Glycerol; *Etoh*: Ethanol; *NADHcon*: NADH consumed; *NADHpro*: NADH produced.

## Competing interests

The authors declare that they have no competing interests.

## Authors' contributions

JP, SG and CB contributed to the metabolic model set-up and flux calculation.

GH carried out the genetic work.

GH, JP, SG, and CB contributed to the fermentations experiments.

SG, EN, CB, and SA conceived of the study, and participated in its design and coordination and helped to draft the manuscript.

All authors read and approved the final manuscript.

## Supplementary Material

Additional file 1 ***S.cerevisiae *metabolic network model**. The Excel sheet "reactions" contains all the reactions in the model categorized by their metabolic pathway, the sheet "abbreviated metabolites" enlists the abbreviations of the metabolites, the sheet "symbolic variables" enlists the variables used for the description of macromolecule composition, the sheet "variables' values" shows for each variable the value used for the resolution of the equation system corresponding to the model. Click here for file

## References

[B1] Eriksson P André L Ansell R Blomberg A Adler L Cloning and characterization of *GPD2*, a second gene encoding *sn*-glycerol 3-phosphate dehydrogenase (NAD^+^) in *Saccharomyces cerevisiae*, and its comparison with *GPD1* Molecular Microbiology 1995 17 1 95 107 10.1111/j.1365-2958.1995.mmi_17010095.x 7476212

[B2] Gancedo C Gancedo JM Sols A Glycerol Metabolism in Yeasts European Journal of Biochemistry 1968 5 2 165 172 10.1111/j.1432-1033.1968.tb00353.x 5667352

[B3] Bjorkqvist S Ansell R Adler L Liden G Physiological response to anaerobicity of glycerol-3-phosphate dehydrogenase mutants of *Saccharomyces cerevisiae* Applied Environmental Microbiology 1997 63 1 128 132 10.1128/aem.63.1.128-132.1997 PMC168310 8979347

[B4] Oura E Reaction products of yeast fermentations Process Biochememistry 1977 12 19 21

[B5] Albers E Larsson C Liden G Niklasson C Gustafsson L Influence of the nitrogen source on *Saccharomyces cerevisiae *anaerobic growth and product formation Applied Environmental Microbiology 1996 62 9 3187 3195 10.1128/aem.62.9.3187-3195.1996 PMC168115 8795209

[B6] van Dijken J Scheffers WA Redox balances in the metabolism of sugars by yeasts FEMS Microbiology Letters 1986 32 199 224

[B7] Ansell R Granath K Hohmann S Thevelein JM Adlerand L The two isoenzymes for yeast NAD^+^-dependent glycerol 3-phosphate dehydrogenase encoded by *GPD1 *and *GPD2 *have distinct roles in osmoadaptation and redox regulation The EMBO journal 1997 16 2179 2187 10.1093/emboj/16.9.2179 9171333 PMC1169820

[B8] Rigoulet M Aguilaniu H Avéret N Bunoust O Camougrand N Grandier-Vazeille X Larsson C Pahlman IL Manon S Gustafsson L Organization and regulation of the cytosolic NADH metabolism in the yeast *Saccharomyces cerevisiae* Molecular and Cellular Biochemistry 2004 256-257 1 73 10.1023/B:MCBI.0000009888.79484.fd 14977171

[B9] Athenstaedt K Daum G Phosphatidic acid, a key intermediate in lipid metabolism European Journal Biochemistry 1999 266 1 1 16 10.1046/j.1432-1327.1999.00822.x 10542045

[B10] Hohmann S Osmotic Stress Signaling and Osmoadaptation in Yeasts Microbiology and Molecular Biology Review 2002 66 2 300 372 10.1128/MMBR.66.2.300-372.2002 PMC120784 12040128

[B11] Blomberg A Adler L Roles of glycerol and glycerol-3-phosphate dehydrogenase (NAD^+^) in acquired osmotolerance of *Saccharomyces cerevisiae* Journal of Bacteriology 1989 171 2 1087 1092 2644223 10.1128/jb.171.2.1087-1092.1989 PMC209705

[B12] Luyten K Albertyn J Skibbe WF Prior BA Ramos J Thevelein JM Hohmann S Fps1, a yeast member of the MIP family of channel proteins, is a facilitator for glycerol uptake and efflux and is inactive under osmotic stress The EMBO Journal 1995 14 1360 1371 7729414 10.1002/j.1460-2075.1995.tb07122.x PMC398221

[B13] Aldiguier AS Alfenore S Cameleyre X Goma G Uribelarrea JL Guillouet SE Molina-Jouve C Synergistic temperature and ethanol effect on *Saccharomyces cerevisiae *dynamic behaviour in ethanol bio-fuel production Bioprocess and Biosystems Engineering 2004 26 4 217 10.1007/s00449-004-0352-6 15098119

[B14] Michnick S Roustan JL Remize F Barre P Dequin S Modulation of Glycerol and Ethanol Yields During Alcoholic Fermentation in *Saccharomyces cerevisiae *Strains Overexpressed or Disrupted for *GPD1 *Encoding Glycerol 3-Phosphate Dehydrogenase Yeast 1997 13 9 783 793 10.1002/(SICI)1097-0061(199707)13:9<783::AID-YEA128>3.0.CO;2-W 9234667

[B15] Valadi H Larsson C Gustafsson L Improved ethanol production by glycerol-3-phosphate dehydrogenase mutants of *Saccharomyces cerevisiae* Applied Microbiology and Biotechnology 1998 50 4 434 10.1007/s002530051317 9830094

[B16] Nissen TL Hamann CW Kielland-Brandt MC Nielsen J Villadsen J Anaerobic and aerobic batch cultivations of *Saccharomyces cerevisiae *mutants impaired in glycerol synthesis Yeast 2000 16 5 463 474 10.1002/(SICI)1097-0061(20000330)16:5<463::AID-YEA535>3.0.CO;2-3 10705374

[B17] Guo Zp Zhang L Ding Zy Wang ZX Shi GY Interruption of glycerol pathway in industrial alcoholic yeasts to improve the ethanol production Applied Microbiology and Biotechnology 2009 82 2 287 10.1007/s00253-008-1777-7 19018525

[B18] Nissen TL Kielland-Brandt MC Nielsen J Villadsen J Optimization of Ethanol Production in *Saccharomyces cerevisiae *by Metabolic Engineering of the Ammonium Assimilation Metabolic Engineering 2000 2 1 69 77 10.1006/mben.1999.0140 10935936

[B19] Bro C Regenberg B Förster J Nielsen J In silico aided metabolic engineering of Saccharomyces cerevisiae for improved bioethanol production Metabolic Engineering 2006 8 2 102 10.1016/j.ymben.2005.09.007 16289778

[B20] Boulahya K Evaluation des potentialités fermentaires de souches mutées de *S. cerevisiae *en vue d'une production nulle de glycérol dans une fermentation éthanolique 2005 Toulouse: Université de Toulouse, INSA

[B21] Kong QX Cao LM Zhang AL Chen X Overexpressing GLT1 in gpd1 Δ mutant to improve the production of ethanol of *Saccharomyces cerevisiae* Applied Microbiology and Biotechnology 2007 73 6 1382 10.1007/s00253-006-0610-4 17021874

[B22] Kong QX Zhang AL Cao LM Chen X Over-expressing GLT1 in a gpd2 Δ mutant of *Saccharomyces cerevisiae *to improve ethanol production Applied Microbiology and Biotechnology 2007 75 6 1361 10.1007/s00253-007-0948-2 17505823

[B23] Zhang A Kong Q Cao L Chen X Effect of *FPS1 *deletion on the fermentation properties of *Saccharomyces cerevisiae* Letters in Applied Microbiology 2007 44 2 212 217 10.1111/j.1472-765X.2006.02041.x 17257263

[B24] Kong QX Gu JG Cao LM Zhang AL Chen X Zhao XM Improved production of ethanol by deleting FPS1 and over-expressing GLT1 in *Saccharomyces cerevisiae* Biotechnology Letters 2006 28 24 2033 10.1007/s10529-006-9185-5 17043906

[B25] Cao L Zhang A Kong Q Xu X Josine TL Chen X Overexpression of GLT1 in fps1D gpdD mutant for optimum ethanol formation by Saccharomyces cerevisiae Biomolecular Engineering 2007 24 6 638 10.1016/j.bioeng.2007.10.003 18032102

[B26] Alfenore S Cameleyre X Benbadis L Bideaux C Uribelarrea JL Goma G Molina-Jouve C Guillouet SE Aeration strategy: a need for very high ethanol performance in *Saccharomyces cerevisiae *fed-batch process Applied Microbiology and Biotechnology 2004 63 5 537 10.1007/s00253-003-1393-5 12879304

[B27] Bideaux C Alfenore S Cameleyre X Molina-Jouve C Uribelarrea JL Guillouet SE Minimization of Glycerol Production during the High-Performance Fed-Batch Ethanolic Fermentation Process in *Saccharomyces cerevisiae*, Using a Metabolic Model as a Prediction Tool Applied Environmental Microbiology 2006 72 3 2134 2140 10.1128/AEM.72.3.2134-2140.2006 PMC1393190 16517663

[B28] Alper H Fischer C Nevoigt E Stephanopoulos G Tuning genetic control through promoter engineering PNAS 2005 102 36 12678 12683 10.1073/pnas.0504604102 16123130 PMC1200280

[B29] Nevoigt E Kohnke J Fischer CR Alper H Stahl U Stephanopoulos G Engineering of Promoter Replacement Cassettes for Fine-Tuning of Gene Expression in *Saccharomyces cerevisiae* Applied Environmental Microbiology 2006 72 8 5266 5273 10.1128/AEM.00530-06 PMC1538763 16885275

[B30] Sambrook J Maniatis T Fritsch EF Molecular cloning: a laboratory manual 1989 2 Cold Spring Harbor, N.Y.: Cold Spring Harbor Laboratory

[B31] Alfenore S Molina-Jouve C Guillouet S Uribelarrea JL Goma G Benbadis L Improving ethanol production and viability of *Saccharomyces cerevisiae *by a vitamin feeding strategy during fed-batch process Applied Microbiology and Biotechnology 2002 60 1 67 10.1007/s00253-002-1092-7 12382043

[B32] Gueldener U Heinisch J Koehler GJ Voss D Hegemann JH A second set of loxP marker cassettes for Cre-mediated multiple gene knockouts in budding yeast Nucleic Acids Res 2002 30 6 e23 10.1093/nar/30.6.e23 11884642 PMC101367

[B33] Gietz RD Schiestl RH Applications of high efficiency lithium acetate transformation of intact yeast cells using single-stranded nucleic acids as carrier Yeast 1991 7 3 253 263 10.1002/yea.320070307 1882550

[B34] Nevoigt E Stahl U Reduced pyruvate decarboxylase and increased glycerol-3-phosphate dehydrogenase [NAD^+^] levels enhance glycerol production in *Saccharomyces cerevisiae* Yeast 1996 12 13 1331 1337 10.1002/(SICI)1097-0061(199610)12:13<1331::AID-YEA28>3.0.CO;2-0 8923738

[B35] Duboc P Stockar Uv Systematic errors in data evaluation due to ethanol stripping and water vaporization Biotechnology and Bioengineering 1998 58 4 428 439 10.1002/(SICI)1097-0290(19980520)58:4<428::AID-BIT10>3.0.CO;2-7 10099277

[B36] Bideaux C Goma G Uribelarrea JL Dahhou B Roux G Stoichiometric modelling approach for microbial cultures monitoring International Journal of Modelling, Identification and Control 2008 3 413 10.1504/IJMIC.2008.020550

[B37] Ryan ED Kohlhaw GB Subcellular Localization of Isoleucine-Valine Biosynthetic Enzymes in Yeast Jounal of Bacteriology 1974 120 2 631 637 10.1128/jb.120.2.631-637.1974 PMC245821 4616942

[B38] Brandriss MC Magasanik B Subcellular compartmentation in control of converging pathways for proline and arginine metabolism in *Saccharomyces cerevisiae* Journal of Bacteriology 1981 145 3 1359 1364 7009582 10.1128/jb.145.3.1359-1364.1981 PMC217140

[B39] Kispal G Steiner H Court DA Rolinski B Lill R Mitochondrial and Cytosolic Branched-chain Amino Acid Transaminases from Yeast, Homologs of the myc Oncogene-regulated Eca39 Protein Journal of Biological Chemistry 1996 271 40 24458 24464 10.1074/jbc.271.40.24458 8798704

[B40] Maaheimo H Fiaux J Cakar ZP Bailey JE Sauer U Szyperski T Central carbon metabolism of *Saccharomyces cerevisiae *explored by biosynthetic fractional 13C labeling of common amino acids European Journal of Biochemistry 2001 268 8 2464 2479 10.1046/j.1432-1327.2001.02126.x 11298766

[B41] Förster J Famili I Fu P Palsson BÃ Nielsen J Genome-Scale Reconstruction of the *Saccharomyces cerevisiae *Metabolic Network Genome Research 2003 13 2 244 253 10.1101/gr.234503 12566402 PMC420374

[B42] Duarte NC Herrgard MJ Palsson BO Reconstruction and Validation of *Saccharomyces cerevisiae *iND750, a Fully Compartmentalized Genome-Scale Metabolic Model Genome Research 2004 14 1298 1309 10.1101/gr.2250904 15197165 PMC442145

[B43] Gombert AK Moreira dos Santos M Christensen B Nielsen J Network Identification and Flux Quantification in the Central Metabolism of *Saccharomyces cerevisiae *under Different Conditions of Glucose Repression Journaml of Bacteriology 2001 183 4 1441 1451 10.1128/JB.183.4.1441-1451.2001 PMC95019 11157958

[B44] Fiaux J Cakar ZP Sonderegger M Wuthrich K Szyperski T Sauer U Metabolic-Flux Profiling of the Yeasts *Saccharomyces cerevisiae *and *Pichia stipitis* Eukaryotic Cell 2003 2 1 170 180 10.1128/EC.2.1.170-180.2003 12582134 PMC141173

[B45] Remize F Cambon B Barnavon L Dequin S Glycerol formation during wine fermentation is mainly linked to Gpd1p and is only partially controlled by the HOG pathway Yeast 2003 20 15 1243 1253 10.1002/yea.1041 14618562

[B46] Olz R Larsson K Adler L Gustafsson L Energy flux and osmoregulation of *Saccharomyces cerevisiae *grown in chemostats under NaCl stress Journal of Bacteriology 1993 175 8 2205 2213 8468281 10.1128/jb.175.8.2205-2213.1993 PMC204505

[B47] Heyland J Fu J Blank LM Correlation between TCA cycle flux and glucose uptake rate during respiro-fermentative growth of *Saccharomyces cerevisiae* Microbiology 2009 mic.0.030213-030210 19684065 10.1099/mic.0.030213-0

[B48] Pahlman Il Larsson C Averet N Bunoust O Boubekeur S Gustafsson L Rigoulet M Kinetic Regulation of the Mitochondrial Glycerol-3-phosphate Dehydrogenase by the External NADH Dehydrogenase in *Saccharomyces cerevisiae* Journal of Biological Chemistry 2002 277 31 27991 27995 10.1074/jbc.M204079200 12032156

[B49] Nevoigt E Stahl U Osmoregulation and glycerol metabolism in the yeast *Saccharomyces cerevisiae* FEMS Microbiology Reviews 1997 21 3 231 241 10.1111/j.1574-6976.1997.tb00352.x 9451815

[B50] Slaughter B Li R Toward a molecular interpretation of the surface stress theory for yeast morphogenesis Current Opinion in Cell Biology 2006 18 1 47 10.1016/j.ceb.2005.11.003 16337116

[B51] Koch A The surface stress theory of microbial morphogenesis Advances in Microbial Physiology 1983 24 301 366 full_text 6364728 10.1016/s0065-2911(08)60388-4

[B52] van Dijken JP Bauer J Brambilla L Duboc P Francois JM Gancedo C Giuseppin MLF Heijnen JJ Hoare M Lange HC An interlaboratory comparison of physiological and genetic properties of four *Saccharomyces cerevisiae *strains Enzyme and Microbial Technology 2000 26 9-10 706 10.1016/S0141-0229(00)00162-9 10862876

